# Corneal confocal microscopy reveals trigeminal small sensory fiber neuropathy in amyotrophic lateral sclerosis

**DOI:** 10.3389/fnagi.2014.00278

**Published:** 2014-10-16

**Authors:** Giulio Ferrari, Enrico Grisan, Fabio Scarpa, Raffaella Fazio, Mauro Comola, Angelo Quattrini, Giancarlo Comi, Paolo Rama, Nilo Riva

**Affiliations:** ^1^Cornea and Ocular Surface Disease Unit, Eye Repair Lab, Istituto di Ricovero e Cura a Carattere Scientifico San Raffaele Scientific InstituteMilan, Italy; ^2^Department of Information Engineering, University of PadovaPadova, Italy; ^3^Department of Neurology and Institute of Experimental Neurology (INSPE), Istituto di Ricovero e Cura a Carattere Scientifico San Raffaele Scientific InstituteMilan, Italy; ^4^Vita e Salute San Raffaele UniversityMilan, Italy

**Keywords:** motor neuron disease, neuropathy, facial-onset sensory and motor neuronopathy, neuroophthalmology, neuromuscular, cornea, ALS

## Abstract

Although subclinical involvement of sensory neurons in amyotrophic lateral sclerosis (ALS) has been previously demonstrated, corneal small fiber sensory neuropathy has not been reported to-date. We examined a group of sporadic ALS patients with corneal confocal microscopy, a recently developed imaging technique allowing *in vivo* observation of corneal small sensory fibers. Corneal confocal microscopy (CCM) examination revealed a reduction of corneal small fiber sensory nerve number and branching in ALS patients. Quantitative analysis demonstrated an increase in tortuosity and reduction in length and fractal dimension of ALS patients’ corneal nerve fibers compared to age-matched controls. Moreover, bulbar function disability scores were significantly related to measures of corneal nerve fibers anatomical damage. Our study demonstrates for the first time a corneal small fiber sensory neuropathy in ALS patients. This finding further suggests a link between sporadic ALS and facial-onset sensory and motor neuronopathy (FOSMN) syndrome, a rare condition characterized by early sensory symptoms (with trigeminal nerve distribution), followed by wasting and weakness of bulbar and upper limb muscles. In addition, the finding supports a model of neurodegeneration in ALS as a focally advancing process.

## Introduction

Amyotrophic lateral sclerosis (ALS) is a fatal disorder primarily characterized by progressive degeneration of upper (UMN) and lower motor neurons (LMN), in the brain and spinal cord (Riva et al., 2011). Despite the fact that a modest age-related decrease of skin nerve fiber density has been observed in both healthy controls and ALS patients (Lauria et al., 2010; Weis et al., 2011), subclinical involvement of sensory neurons in the neuro-degenerative process has also been independently demonstrated by previous clinical and pathologic studies (Kawamura et al., 1981; Heads et al., 1991; Hammad et al., 2007; Weis et al., 2011). Moreover, a link between ALS and facial-onset sensory and motor neuronopathy (FOSMN) syndrome, a rare condition characterized by early sensory symptoms (with trigeminal nerve distribution) followed by wasting and weakness of bulbar and upper limb muscles, has also been suggested recently (Fluchere et al., 2011; Dalla Bella et al., 2014). The aim of this study was therefore to examine a group of sporadic ALS patients with corneal confocal microscopy (CCM), a recently developed imaging technique allowing *in vivo* observation of corneal small sensory fibers. Corneal confocal microscopy has a number of comparative advantages over previous techniques such as skin biopsy, allowing non invasive, potentially repeatable and quantitative analysis of small sensory fibers at the microscopic level (Ferrari et al., 2010).

## Methods

### Patients

In this study, which was approved by the local Ethic Committee, eight patients with sporadic ALS (mean age = 67 years, standard deviation (SD) = 5 years) were recruited consecutively, upon obtaining written informed consent. Seven age-matched healthy individuals served as controls (mean age = 60 years; SD = 13 years; Table 1). Patients with a clinical diagnosis of laboratory-supported probable, probable and definite ALS, according to revised Escorial criteria, were deemed eligible for the study (Brooks et al., 2000). Exclusion criteria were: ocular surface diseases, contact lens use, eye drop treatment and significant concomitant medical or neurological diseases, including respiratory failure and diabetes. Patients were graded in terms of UMN “burden”, by totalling the number of pathological UMN signs on examination (UMN score: range: 0–16). Muscle strength was graded on the Medical Research Council (MRC) scale (from 0–5) in selected upper and lower limb muscle groups (range: 0–120). Disease severity was assessed using the ALS Functional Rating Scale-revised (ALS-FRS-r) and the ALS Severity Score (ALS-SS); the ALS-FRS-r and ALS-SS bulbar sub-scores were then calculated. The rate of disease progression was calculated using the following formula: Disease progression rate = (48−ALS-FRS-r score)/disease duration (Riva et al., 2012). Corneal sensitivity was measured with a Cochet-Bonnet corneal esthesiometer, as described (Roszkowska et al., 2004).

**Table 1:** **Study patients characteristics**.

	ALS patients (*N*: 8)	Controls (*N*: 7)
**Sex (M/F)**	4/4	2/5
**Age (years)**	67.2 (5)	60.1 (13)
**Disease duration (months)**	19.6 (9.9)	n.a
**UMN score**	11.1 (5.2)	n.a
**MRC sum score**	82.3 (35.9)	n.a
**ALS-FRS-r**	30.5 (9.2)	n.a
**ALS-FRS-r bulbar score**	9.2 (2.5)	n.a
**ALS-SS**	25.6 (6.9)	n.a
**ALS-SS bulbar score**	14.9 (4.1)	n.a
**Corneal sensibility**	56.8 (5.4)	58.9 (2.8)
**CNL (µm)**	1784 (414)	2284 (369)
**CNT** (**µm**^**-1**^)	1.30 (0.35)	0.71 (0.28)
**CNFD**	1.24 (0.04)	1.19 (0.09)

*Mean and (Standard Deviation) values are shown. ALS: Amyotrophic Lateral Sclerosis. UMN: Upper Motor Neuron; MRC: Medical Research Council Sum Score; ALS-FRS-r: ALS Functional Rating Scale-revised; ALS-SS: ALS Severity Score; CNL: Corneal Nerves Total Length; CNT: Corneal Nerves Mean Tortuosity; CNFD: Corneal Nerves Fractal Dimension*.

### Corneal confocal microscopy

Patients were examined with an *in vivo* laser CCM (Heidelberg Retina Tomograph II with Rostock Cornea Module; Heidelberg Engineering GmbH, Heidelberg, Germany) and the sub-basal nerve plexus was imaged, as previously described (Gemignani et al., 2010) and shown in detail by Tavakoli (Tavakoli and Malik, 2011). A minimum of six images of the sub-epithelial plexus in each eye were randomly selected; the examiner (GF) was masked with respect to the diagnosis. Images were then automatically processed using a previously published algorithm, providing the identification of the nerves and their branching (Scarpa et al., 2008). The following corneal nerve parameters were computed: total length (CNL, i.e., the total length of corneal nerves), fractal dimension using the box-counting approach (CNFD, i.e., quantification of the nerve structure complexity) and mean tortuosity (CNT, i.e., estimate of the straying of the shape of the corneal nerves from a smooth line) (Grisan et al., 2008).

### Statistical analysis

Between group differences were assessed with the Mann-Whitney test. In ALS patients, correlation between clinical and CCM data was investigated with Spearman’s rank correlation (r). Statistical significance was considered at *p* < 0.05. All tests were performed using SPSS software (Technologies, Inc., Chicago, IL, USA).

## Results

**Figure 1:**
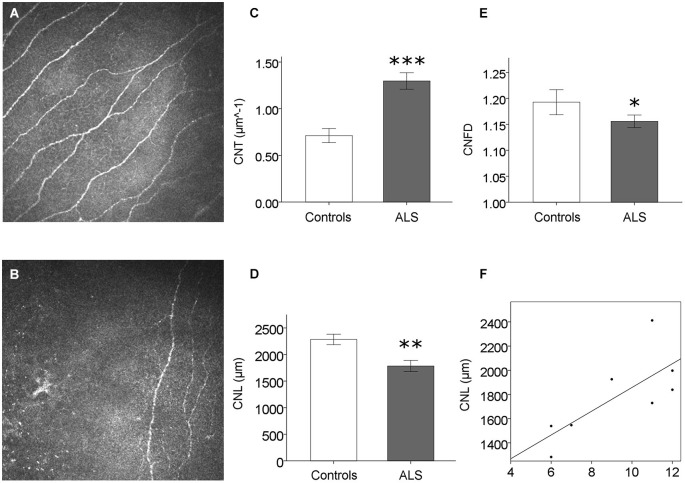
**Corneal small fiber sensory neuropathy in ALS. (A)** and **(B)** exemplificative CCM frames: the reduction of corneal small sensory nerve fiber number and branching is evident in ALS patients **(B)** compared to controls **(A)**, and confirmed by CCM quantitative analysis **(C–E)**. **(F)** Significant correlation between CNL (a measure of corneal nerve damage) and ALS-FRS bulbar score (*r* = 0.764, *p* = 0.027). *p < 0.05; **p < 0.005; ***p < 0.0005.

No sensory symptoms or signs could be detected in ALS patients. Corneal sensitivity did not differ between ALS patients and controls (Table 1). Corneal confocal microscopy examination, however, revealed a reduction of corneal small fiber sensory nerve number and branching in ALS patients (representative confocal microscopy pictures from controls and ALS patients are shown in Figures 1A,B, respectively). At quantitative analysis, CNT was significantly higher in ALS patients (mean = 1.30, SD = 0.35 µm^−1^) compared with controls (mean = 0.71, SD = 0.28 µm^−1^; *p* < 0.0005, Figure 1C). On the contrary, CNL (Figure 1D) and CNFD (Figure 1E) were significantly lower in ALS patients (CNL: mean = 1784 µm, SD = 414 µm; CNFD: mean = 1.24, SD = 0.04) compared with controls (CNL: mean = 2284 µm, SD = 369 µm; CNFD: mean = 1.19, SD = 0.09), (*p* = 0.004 and *p* < 0.011, respectively). These data suggest that a corneal sensory neuropathy exists in ALS patients. Amyotrophic lateral sclerosis-Functional Rating Scale-bulbar score was significantly related to CNL (*r* = 0.764, *p* = 0.027) and CNFD (*r* = 0.715, *p* = 0.046); coherently, ALS-SS-bulbar score was significantly related to both CNL (*r* = 0.908, *p* = 0.002) and CNFD (*r* = 0.847, *p* = 0.008) (Figure 1F, Table 2).

**Table 2:** **Correlation analysis between clinical and confocal microscopy data in ALS patients**.

Clinical data	Confocal microscopy		
	CNL	CNT	CNFD
**Age**	*r* = −0.452 *p* = 0.260	*ρ* = 0.476 *P* = 0.233	*ρ* = −0.357 *P* = 0.385
**Disease duration**	*r* = −0.263 *P* = 0.528	*ρ* = −0.491 *P* = 0.217	*ρ* = −0.383 *P* = 0.349
**MRC sum score**	*r* = −0.204 *P* = 0.629	*P* = 0.263 *P* = 0.528	*ρ* = −0.228 *P* = 0.588
**UMN score**	*ρ* = −0.196 *P* = 0.641	*P* = 0.147 *P* = 0.728	*ρ* = −0.086 *P* = 0.840
**ALS-FRS**	*ρ* = 0.095 *P* = 0.823	*ρ* = 0.238 *P* = 0.570	*ρ* = 0.119 *P* = 0.779
**ALS-FRS-bulbar score**	***ρ* = 0.764** ***P* = 0.027**	*ρ* = 0.158 *P* = 0.709	***ρ* = 0.715** ***P* = 0.046**
**ALS SS**	*ρ* = 0.293 *P* = 0.482	*ρ* = 0.220 *P* = 0.601	*ρ* = 0.293 *P* = 0.482
**ALS SS bulbar score**	***ρ* = 0.908** ***P* = 0.002**	*ρ* = 0.086 *P* = 0.840	***ρ* = 0.847** ***P* = 0.008**
**Disease progression rate**	*ρ* = −0.143 *P* = 0.736	*ρ* = −0.095 *P* = 0.823	*ρ* = −0.095 *P* = 0.823
**Corneal sensitivity**	*ρ* = 0.299 *P* = 0.515	*ρ* = 0.478 *P* = 0.278	*ρ* = 0.299 *P* = 0.515

***ρ:** Spearmann Correlation Coefficient; ALS: Amyotrophic Lateral Sclerosis. UMN: Upper Motor Neuron; MRC: Medical Research Council Sum Score; ALS-FRS-r: ALS Functional Rating Scale-revised; ALS-SS: ALS Severity Score; CNL: Corneal Nerves Total Length; CNT: Corneal Nerves Mean Tortuosity; CNFD: Corneal Nerves Fractal Dimension*.

## Discussion

Our study demonstrates for the first time a corneal small fiber sensory neuropathy in sporadic ALS patients, consistent with previous clinical, pathological and neurophysiological studies showing subclinical sensory neuron involvement in this disease and confirming that neurodegeneration exceeds the neuronal system upon which clinical diagnosis relies (Hammad et al., 2007; Isaacs et al., 2007; Weis et al., 2011). Mild sensory symptoms and signs have been reported in a percentage ranging from 1% to 32% of sporadic ALS patients (Hammad et al., 2007). Evidence of sensory nerve fibers pathology has been confirmed by both sural nerve biopsy studies and autopsy studies, suggesting the preferential vulnerability of large axon and dorsal root ganglia neurons (Kawamura et al., 1981; Heads et al., 1991). However, more recently, a small-fiber neuropathy has been demonstrated in up to 79% of ALS patient skin biopsies (Weis et al., 2011).

Interestingly, the hypothesis that FOSMN syndrome represents an unusual ALS phenotype has been recently proposed (Dalla Bella et al., 2014). Facial-onset sensory and motor neuronopathy hallmarks are the development of sensory symptoms within the face followed by evolution of sensory deficits and signs of LMN degeneration of bulbar and upper limb muscles, such as fasciculations, cramps, muscle weakness and wasting. The hypothesis of a neurodegenerative pathogenesis has also been proposed (Vucic et al., 2012). However, the notion that FOSMN syndrome represents an unusual ALS phenotype is still object of debate (Vucic, 2014). Although no CNN systematic studies have been performed in FOSMN yet, we speculate that our findings may further suggest a link between this rare condition and sporadic ALS.

Our results also show that in ALS patients corneal nerve fiber damage was significantly correlated with bulbar district disability scores, but not with age, UMN or LMN involvement, disease duration or spinal disability. This finding may be correctly viewed in the light of the recent model of neurodegeneration in ALS as a focally advancing process (Ravits et al., 2007).

Although studies on larger cohorts are needed to define corneal small fiber sensory neuropathy in different stages of disease or subgroup of patients, this study confirms subclinical sensory neuron (trigeminal) involvement in sporadic ALS, contributing to the understanding of the pathomechanisms of this disease.

## Conflict of interest statement

The authors declare that the research was conducted in the absence of any commercial or financial relationships that could be construed as a potential conflict of interest.
